# Skin Lesion Analysis towards Melanoma Detection Using Deep Learning Network

**DOI:** 10.3390/s18020556

**Published:** 2018-02-11

**Authors:** Yuexiang Li, Linlin Shen

**Affiliations:** 1 Computer Vision Institute, College of Computer Science and Software Engineering, Shenzhen University, Shenzhen 518060, China; yuexiang.li@szu.edu.cn; 2 Guangdong Key Laboratory of Intelligent Information Processing, Shenzhen University, Shenzhen 518060, China

**Keywords:** skin lesion classification, melanoma recognition, deep convolutional network, fully-convolutional residual network

## Abstract

Skin lesions are a severe disease globally. Early detection of melanoma in dermoscopy images significantly increases the survival rate. However, the accurate recognition of melanoma is extremely challenging due to the following reasons: low contrast between lesions and skin, visual similarity between melanoma and non-melanoma lesions, etc. Hence, reliable automatic detection of skin tumors is very useful to increase the accuracy and efficiency of pathologists. In this paper, we proposed two deep learning methods to address three main tasks emerging in the area of skin lesion image processing, i.e., lesion segmentation (task 1), lesion dermoscopic feature extraction (task 2) and lesion classification (task 3). A deep learning framework consisting of two fully convolutional residual networks (FCRN) is proposed to simultaneously produce the segmentation result and the coarse classification result. A lesion index calculation unit (LICU) is developed to refine the coarse classification results by calculating the distance heat-map. A straight-forward CNN is proposed for the dermoscopic feature extraction task. The proposed deep learning frameworks were evaluated on the ISIC 2017 dataset. Experimental results show the promising accuracies of our frameworks, i.e., 0.753 for task 1, 0.848 for task 2 and 0.912 for task 3 were achieved.

## 1. Introduction

Melanoma is the most deadly form of skin cancer and accounts for about 75% of deaths associated with skin cancer [1]. Accurate recognition of melanoma in early stage can significantly increase the survival rate of patients. However, the manual detection of melanoma produces huge demand of well-trained specialists, and suffers from inter-observer variations. A reliable automatic system for melanoma recognition, increasing the accuracy and efficiency of pathologists, is worthwhile to develop.

The dermoscopy technique has been developed to improve the diagnostic performance of melanoma. Dermoscopy is a noninvasive skin imaging technique of acquiring a magnified and illuminated image of skin region for increased clarity of the spots [2], which enhances the visual effect of skin lesion by removing surface reflection. Nevertheless, automatic recognition of melanoma from dermoscopy images is still a difficult task, as it has several challenges. First, the low contrast between skin lesions and normal skin region makes it difficult to segment accurate lesion areas. Second, the melanoma and non-melanoma lesions may have high degree of visual similarity, resulting in the difficulty for distinguishing melanoma lesion from non-melanoma. Third, the variation of skin conditions, e.g., skin color, natural hairs or veins, among patients produce different appearance of melanoma, in terms of color and texture, etc.

Skin lesion segmentation is the essential step for most classification approaches. Recent review of automated skin lesion segmentation algorithms can be found in [3]. Accurate segmentation can benefit the accuracy of subsequent lesion classification. Extensive studies [4,5,6,7,8,9,10,11,12] have been made to produce decent lesion segmentation results. For example, Gomez et al. proposed an unsupervised algorithm, named Independent Histogram Pursuit (IHP), for the segmentation of skin lesion [13]. The algorithm was tested on five different dermatological datasets, and achieved a competitive accuracy close to 97%. Zhou developed several mean-shift-based approaches for segmenting skin lesions in dermoscopic images [14,15,16]. Garnavi et al. proposed an automated segmentation approach for skin lesion using optimal color channels and hybrid thresholding technique [17]. In more recent research, Pennisi et al. employed Delaunay Triangulation to extract binary masks of skin lesion regions, which does not require any training stage [18]. Ma proposed a novel deformable model using a newly defined speed function and stopping criterion for skin lesion segmentation, which is robust against noise and yields effective and flexible segmentation performance [19]. Yu used a deep learning approach, i.e., a fully convolutional residual network (FCRN), for skin lesion segmentation in dermoscopy images [20].

Based on the segmentation results, hand-crafted features can be extracted for melanoma recognition. Celebi et al. extracted several features, including color and texture from segmented lesion region for skin lesion classification [21]. Schaefer used an automatic border detection approach [22] to segment the lesion area and then assembled the extracted features, i.e., shape, texture and color, for melanoma recognition [23]. On the other hand, some investigations [24] have attempted to directly employ hand-crafted features for melanoma recognition without a segmentation step. Different from approaches using hand-crafted features, deep learning networks use hierarchical structures to automatically extract features. Due to the breakthroughs made by deep learning in an increasing number of image-processing tasks [25,26,27,28], some research has started to apply deep learning approaches for melanoma recognition. Codella et al. proposed a hybrid approach, integrating convolutional neural network (CNN), sparse coding and support vector machines (SVMs) to detect melanoma [29]. In recent research, Codella and his colleagues established a system combining recent developments in deep learning and machine learning approaches for skin lesion segmentation and classification [30]. Kawahara et al. employed a fully convolutional network to extract multi-scale features for melanoma recognition [31]. Yu et al. applied a very deep residual network to distinguish melanoma from non-melanoma lesions [20].

Although lots of work has been proposed, there is still a margin of performance improvement for both skin lesion segmentation and classification. The International Skin Imaging Collaboration (ISIC) is a cooperation focusing on the automatic analysis of skin lesion, and has continuously expanded its datasets since 2016. In ISIC 2017, annotated datasets for three processing tasks related to skin lesion images, including lesion segmentation, dermoscopic feature extraction and lesion classification, were released for researchers to promote the accuracy of automatic melanoma detection methods. Different from the extensively studied lesion segmentation and classification, dermoscopic feature extraction is a new task in the area. Consequently, few studies have been proposed to address the problem.

In this paper, we proposed deep learning frameworks to address the three main processing tasks of skin lesion images proposed in ISIC 2017. The main contribution of this paper can be summarized as follows:

(1) Existing deep learning approaches commonly use two networks to separately perform lesion segmentation and classification. In this paper, we proposed a framework consisting of multi-scale fully-convolutional residual networks and a lesion index calculation unit (LICU) to simultaneously address lesion segmentation (task 1) and lesion classification (task 3). The proposed framework achieved excellent results in both tasks. Henceforth, the proposed framework is named as Lesion Indexing Network (LIN).
(2) We proposed a CNN-based framework, named Lesion Feature Network (LFN), to address task 2, i.e., dermoscopic feature extraction. Experimental results demonstrate the competitive performance of our framework. To the best of our knowledge, we are not aware of any previous work proposed for this task. Hence, this work may become the benchmark for the following related research in the area.
(3) We made detailed analysis of the proposed deep learning frameworks in several respects, e.g., the performances of networks with different depths; and the impact caused by adding different components (e.g., batch normalization, weighted softmax, etc.). This work provides useful guidelines for the design of deep learning networks in related medical research.

## 2. Methods

In this section, we introduce the deep learning methods developed for different tasks.

### 2.1. Lesion Segmentation and Classification (Task 1 & 3)

#### 2.1.1. Pre-Processing

**Figure 1:**
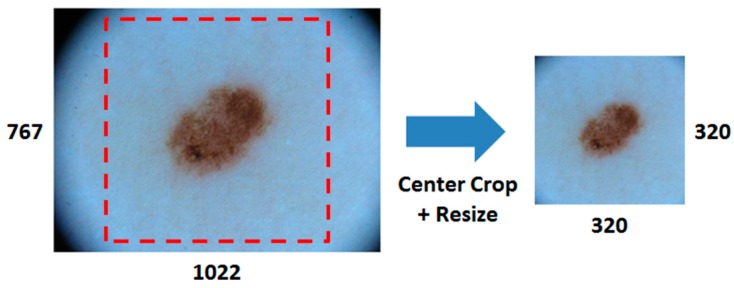
Pre-processing for skin lesion image. First crop the center area and then proportionally resize to a lower resolution. (The numbers of image size are measured by pixels).

The original training set contains 2000 skin lesion images of different resolutions. The resolutions of some lesion images are above 1000 × 700, which require a high cost of computation. It is necessary to rescale the lesion images for the deep learning network. As directly resizing images may distort the shape of the skin lesion, we first cropped the center area of lesion image and then proportionally resize the area to a lower resolution. The size of the center square was set to be 0.8 of the height of the image, and automatically cropped with reference to the image center. As illustrated in Figure 1, this approach not only enlarges the lesion area for feature detection, but also maintains the shape of the skin lesion.

#### 2.1.2. Data Augmentation

The dataset contains three categories of skin lesion, i.e., Melanoma, Seborrheic keratosis and Nevus. As the number of images of different categories varies widely, we accordingly rotated the images belonging to different classes to establish a class-balanced dataset. The dataset augmented with this step is denoted as DR. The number of images of original training set and DR are listed in Table 1. The numbers in the brackets after the category names are the angles for each rotation.

The images in DR are randomly flipped along the *x* or *y*-axis to establish another pair dataset, called DM. The two datasets are separately used to train FCRNs.

#### 2.1.3. Lesion Indexing Network (LIN)

##### Network Architecture

**Figure 2:**
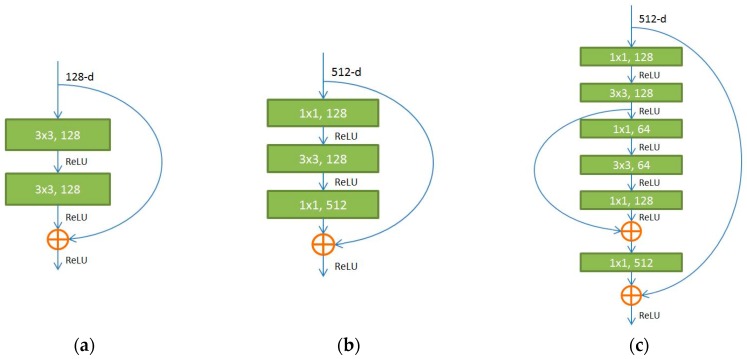
Residual building blocks. (**a**) Plain identity shortcut; (**b**) Bottleneck; (**c**) Residual in Residual (RiR). (**a**,**b**) are adopted in the original FCRN-50 and FCRN-101.

The fully convolutional residual network, i.e., FCRN-88, proposed in our previous work [32], which outperforms the FCRN-50 and FCRN-101 [33], was extended to simultaneously address the tasks of lesion segmentation and classification in this paper. In the previous work [32], a novel residual in residual module (Figure 2c) is proposed to replace the original residual module (Figure 2a,b) to better address the gradient vanishing problem as the network goes deeper. Using the RiR module, the original FCRN-50 was transformed to a deeper model, i.e., FCRN-88. The improved FCRN-88 achieves new state-of-the-art results for the segmentation of HEp-2 specimen images.

**Figure 3:**
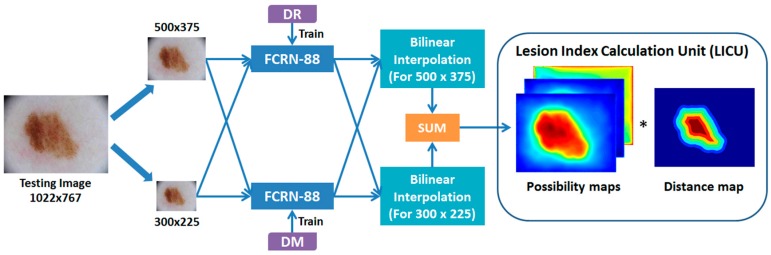
Flowchart of the Lesion Indexing Network (LIN). The framework contains two FCRN and a calculation unit for lesion index. (The numbers of image size are measured by pixels).

Based on FCRN-88, we construct a Lesion Indexing Network (LIN) for skin lesion image analysis. The flowchart of LIN is presented in Figure 3. Two FCRNs trained with datasets using different data augmentation methods are involved. The lesion index calculation unit (LICU) is designed to refine the probabilities for *Melanoma*, *Seborrheic keratosis* and *Nevus*.

In the testing stage, as the fully convolutional network accepts inputs with different sizes, we proportionally resize the skin lesion images to two scales, i.e., ~300 × 300 and ~500 × 500, and send them to the FCRNs, respectively. The results of different scales are interpolated to the original resolution of testing image and sum up to yield the coarse possibility maps. The LICU employs a distance map representing the importance of each pixel to refine the coarse possibilities of skin lesions.

The reason for using separate FCRN-88 trained on different datasets, i.e., DR and DM, is that we found ‘mirror’ operation seems to fool the FCRN-88 during training. The segmentation and classification accuracies on the validation set verified our findings, i.e., the separate network provides better segmentation and classification performance than that of a single FCRN-88 trained on DR + DM.

##### Lesion Index Calculation Unit (LICU)

As the accurate possibility maps of different lesion categories of skin lesion image provide useful information for pathologists, we proposed a component, named Lesion Index Calculation Unit (LICU), to refine the coarse skin lesion possibilities maps from FCRNs.

First, the coarse possibility maps after summation need to be normalized to [0, 1]. Let $v_{i}\left(x,y\right)$ be the value of (*x*, *y*) in *i*th coarse map, the normalized possibility for skin lesions ($p_{i}$) can be deduced by:

(1) $$p_{i}\left(x,y\right)=\frac{v_{i}\left(x,y\right)-\underset{i\in 1,\;2,\;3}{\min}\left(v_{i}\left(x,y\right)\right)}{\sum_{i=1}^{3}\left(v_{i}\left(x,y\right)-\underset{i\in 1,2,3}{\min}\left(v_{i}\left(x,y\right)\right)\right)}\;i\in 1,\;2,\;3$$

**Figure 4:**
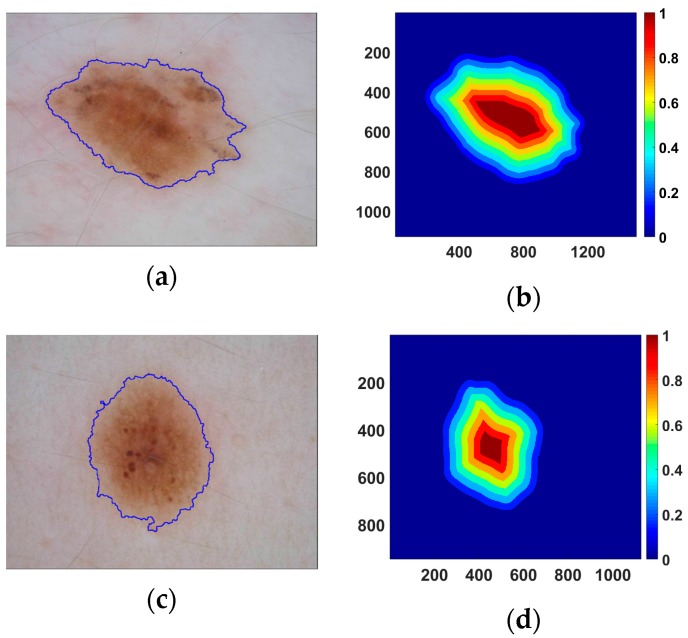
Examples of skin lesion images with outlines (blue) and distance maps. The first column (**a**,**c**) shows the original lesion images and the second (**b**,**d**) shows the corresponding distance maps. The scales for the original lesion images are about 1300 pixels × 1000 pixels and 1000 pixels × 800 pixels, respectively. The numbers of image size of distance maps are measured by pixels. The numbers in color-bar represent corresponding weights.

Each pixel in the lesion area has a different importance for lesion classification. It can be observed from Figure 4a,c that the area near the lesion border of some skin lesion images has a more similar appearance, i.e., color/texture, to skin than that of the center area. The blue lines in Figure 4a,c are the borders of lesions produced by LIN. The lesion area with similar features to skin may provide less information for lesion recognition. Hence, the distances from pixels to the nearest border are used to represent the importance of pixels for lesion classification. Examples of distance maps are shown in the Figure 4b,d. The colors in the distance map represent the weights for corresponding pixels. The distance map is multiplied to each of the normalized coarse possibility maps to generate refined maps. Finally, we average the possibilities in the lesion area of refined maps to obtain the indexes for different categories of skin lesion.

##### Implementation

The proposed LIN is established using *MatConvNet* toolbox [34]. While 80% of the training dataset is used for training, the remainder is used for validation. The FCRNs were individually trained with a mini-batch size of 128 on one GPU (GeForce GTX TITAN X, 12 GB RAM). The details of the training setting are the same as [32]. We stopped the network training early, after 6 epochs, to overcome the overfitting problem.

### 2.2. Dermoscopic Feature Extraction (Task 2)

Dermoscopic feature extraction is a new task announced in ISIC 2017, which aims to extract clinical features from dermoscopic images. Little previous work has addressed this task. In this section, we introduce a CNN-based approach, i.e., the Lesion Feature Network (LFN), developed to address the challenge.

#### 2.2.1. Superpixel Extraction

**Figure 5:**
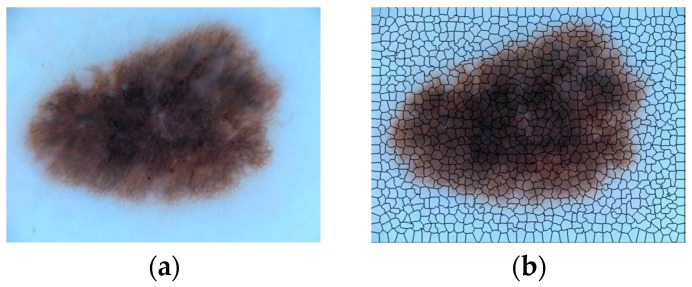
Example of superpixels. The original image (**a**) was subdivided into 996 pieces of superpixel areas (**b**) separated by black lines. The scale for the lesion image is 1022 pixels × 767 pixels.

The ISIC dermoscopic images contain four kinds of dermoscopic features, i.e., Pigment Network (PN), Negative Network (NN), Streaks (S) and Milia-like Cysts (MC). To locate the positions of dermoscopic features, the dermoscopic images were subdivided into superpixels using algorithm introduced in [35]. An example is shown in Figure 5. The original skin lesion image (Figure 5a) was divided into 996 superpixel areas (Figure 5b), which are separated by black lines.

Each superpixel area can be classified into one of five categories: four kinds of dermoscopic features and background (B). Hence, the problem of feature extraction is converted to the classification of superpixel areas. We extract the content of each superpixel according to [35] and resize them to a uniform size, i.e., 56 × 56, for the proposed Lesion Feature Network.

#### 2.2.2. Data Augmentation

The extracted patch dataset is extremely imbalanced. Most of patches only contain the background information. Hence, data augmentation processing is needed to balance the number of images of different categories. Two processing techniques, i.e., Random sample and Patch rotation, were adopted. The number of images of the original and augmented patch datasets is listed in Table 2.

##### Random Sample

As listed in Table 2, the volume of the original background patches is much larger than that of other categories. However, most of background patches contain similar contents. Hence, background patches contain lots of redundant information. To remove the redundancy and decrease the patch volume, the background patches for LFN training are randomly selected from the original patch dataset, which ultimately formed a set of 87,089 background patches.

Due to the extremely large volume of Pigment Network (PN) in the original patch dataset, random sample operation was also applied to PN, resulting in a set of 77,325 PN patches.

##### Patch Rotation

The volumes of NN, MC and S patches are relatively small in the original dataset. Image rotation is employed to augment the volumes. Three angles, i.e., 90, 180 and 270, were adopted for patch rotation, which increases the patch volumes to 12,908, 18,424 and 8324 for NN, MC and S, respectively.

#### 2.2.3. Lesion Feature Network (LFN)

**Figure 6:**
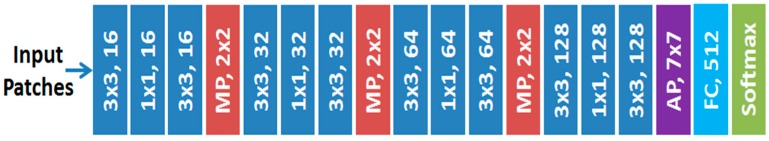
Flowchart of Lesion Feature Network (LFN).

The augmented training set was used to train our Lesion Feature Network (LFN), whose architecture is presented in Figure 6.

While the blue rectangles represent the convolutional layers, the numbers represent kernel size and number of kernels. LFN involves 12 convolutional layers for feature extraction, which can be separated into 4 stages, i.e., 3 convolutional layers per stage. As the 1 × 1 convolution can integrate the features extracted by 3 × 3 convolution for better feature representation, a network in network like structure [36] is adopted for each stage. FC is the fully connected layer. Both max pooling (MP) and average pooling (AP) are used, and the network was trained with softmax loss, defined in (2).

(2) $$L=\frac{1}{N}\sum_{i}L_{i}=\frac{1}{N}\sum_{i}-\log(\frac{e^{f_{y_{i}}}}{\sum_{j}e^{f_{j}}})$$

where $f_{j}$ denotes the *j*-th element (j∈[1,K], *K* is the number of classes) of vector of class scores ***f***, $y_{i}$ is the label of *i*-th input feature and *N* is the number of training data.

Although the data augmentation operation was performed, the obtained training dataset is still imbalanced. To address the problem, weights are assigned for different classes while calculating the softmax loss, to pay more attention to the classes with fewer samples. According to the number of images in the augmented training set, the weights are set to 1, 1, 5, 3 and 8 for B, PN, NN, MC and S, respectively.

#### 2.2.4. Implementation

The proposed LFN is developed using *Keras* toolbox. The patch dataset is separated into the training set and the validation set according to the percentages of 80:20, respectively. The network is optimized by Stochastic Gradient Descent (SGD) [37] with an initial learning rate of 0.01 and a momentum of 0.9. The learning rate decreases with gamma = 0.1. The network was trained on a single GPU (GeForce GTX TITAN X, 12GB RAM) and was observed to converge after 10 epochs of training.

## 3. Performance Analysis

### 3.1. Datasets

**Figure 7:**
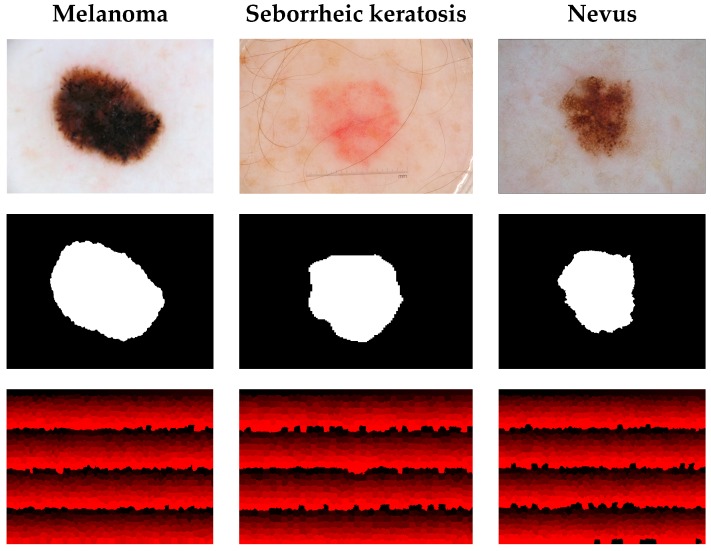
Examples of lesion images from ISIC 2017 and their masks. **The first row** shows the original images of different lesions. **The second row** shows the segmentation masks. **The third row** shows the superpixel mask for dermoscopic feature extraction. The scales for the lesion images are 1022 pixels × 767 pixels, 3008 pixels × 2000 pixels and 1504 pixels × 1129 pixels, respectively.

We use the publicly available *International Skin Imaging Collaboration* (ISIC) 2017 dataset [38] for experiments in this paper. ISIC 2017 provides 2000 skin lesion images as a training set with masks for segmentation, superpixel masks for dermoscopic feature extraction and annotations for classification. The lesion images are classified into three categories, Melanoma, Seborrheic keratosis and Nevus. Melanoma is a malignant skin tumor, which leads to high death rate. The other two kinds of lesion, i.e., Seborrheic keratosis and Nevus, are the benign skin tumors derived from different cells. Figure 7 presents the lesion images from ISIC 2017 and their masks for different tasks. The first row in Figure 7 shows the original skin lesion images. The second row shows the masks for lesion segmentation, while the third row shows the superpixel masks for dermoscopic feature extraction. ISIC 2017 also provides a publicly available validation set with another 150 skin lesion images for evaluation.

In this section, we analyze the performances of the proposed LIN and LFN on the ISIC 2017 validation set. The comparison with benchmark algorithms will be presented in the next section.

### 3.2. Evaluation Metrics

#### 3.2.1. Lesion Segmentation

The ISIC recommends several metrics for performance evaluation, which includes accuracy (*AC*), Jaccard Index (*JA*), Dice coefficient (*DI*), sensitivity (*SE*) and specificity (*SP*). Let $N_{tp}$, $N_{tn}$, $N_{fp}$ and $N_{fn}$ represent the number of true positive, true negative, false positive and false negative, respectively. The criteria can be defined as:

(3) $$AC=\frac{N_{tp}+N_{tn}}{N_{tp}+N_{fp}+N_{tn}+N_{fn}},$$

(4) $$JA=\frac{N_{tp}}{N_{tp}+N_{fn}+N_{fp}},\;DI=\frac{2\times N_{tp}}{2\times N_{tp}+N_{fn}+N_{fp}},$$

(5) $$SE=\frac{N_{tp}}{N_{tp}+N_{fn}},\;SP=\frac{N_{tn}}{N_{tn}+N_{fp}}$$

In this paper, we mainly used the *JA* metric for the evaluation of segmentation performance. The other metrics are measured as reference.

#### 3.2.2. Dermoscopic Feature Extraction and Lesion Classification

The same evaluation metrics, i.e., *AC*, *SE* and *SP*, are employed to assess the performance of dermoscopic feature extraction and lesion classification. Average precision (*AP*), defined in [38], is also involved. In this paper, the primary metric for these two tasks is the area under the ROC curve, i.e., AUC, which is generated by evaluating the true positive rate (TPR), i.e., *SE*, against the false positive rate (*FPR*), defined in (6), at various threshold settings.

(6) $$FPR=\frac{N_{fp}}{N_{tn}+N_{fp}}=1-SP$$

### 3.3. Lesion Indexing Network (LIN)

#### 3.3.1. The Performance on Lesion Segmentation

**Figure 8:**
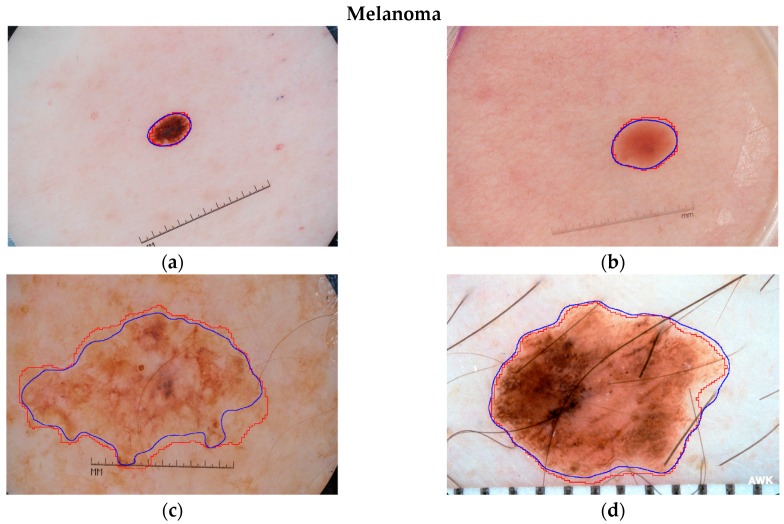

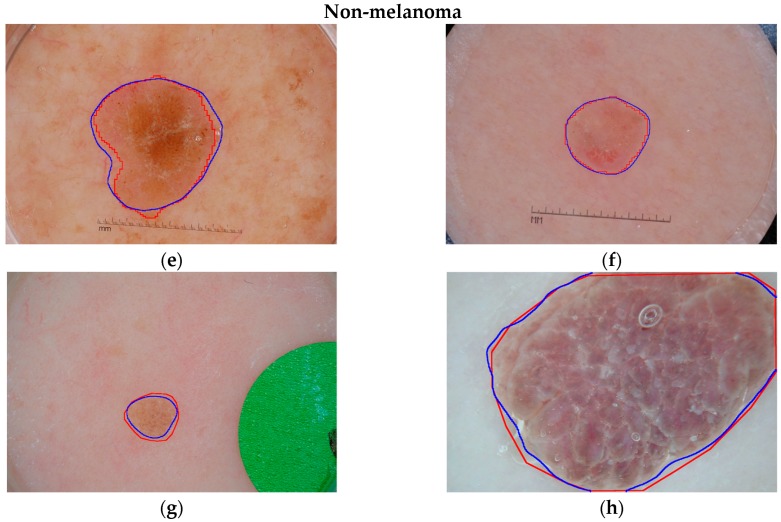
Examples of skin lesion segmentation results produced by LIN for ISIC 2017 validation set. (**a**–**d**) are the results of Melanoma, while (**e**–**h**) are the results for Seborrheic keratosis and Nevus. The blue and red lines represent the segmentation results and ground truths.

To visually analyze the segmentation performance of the proposed LIN, some examples of its segmentation results are presented in Figure 8. The blue and red lines represent the segmentation outlines of LIN and the ground truths, respectively. The examples illustrate some primary challenges in the area of skin lesion image processing. The contrast between lesion and skin region is low in Figure 8b,c,f. Human hair near the lesion region of Figure 8d may influence the segmentation. The artificial scale measure in Figure 8a–c,e,f is another kind of noise information for lesion segmentation. Nevertheless, it can be observed from Figure 8 that the proposed Lesion Indexing Network yields satisfactory segmentation results for all of the challenging cases.

##### Training with DR and DM

**Figure 9:**
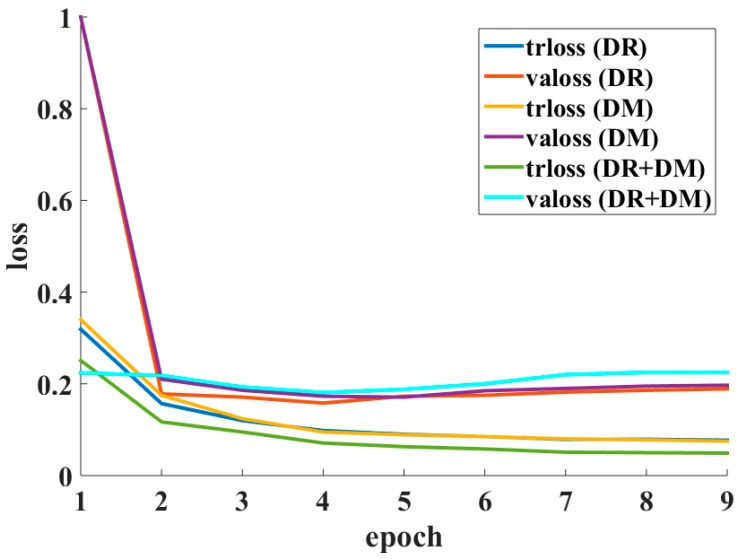
Loss curves of LIN trained with DR, DM and DR + DM.

In the experiments, ‘rotation’ and ‘mirror’ operations were adopted to enlarge the training dataset for Lesion Indexing Network. However, the FCRN-88 seems to be fooled by the ‘mirror’ operation. Figure 9 shows the loss curves of FCRN-88 trained with DR, DM and DR + DM, respectively. Note that ‘trloss’ represents the training loss and ‘valoss’ represents the validation loss.

The validation loss of FCRN-88 trained on DR/DM is stable around 0.2. In contrast, the loss of FCRN-88 trained by DR + DM decreases to about 0.18 and then gradually increases to over 0.2. The FCRN-88 trained with DR + DM has the lowest training loss (green line) but the highest validation loss (cyan line) among the frameworks. This is because the samples of DR and DM are paired. The similar appearances of paired samples make the very deep FCRN-88 easily overfitted to the dataset.

Table 3 listed the JA of single FCRN-88 trained on DR/DR + DM and our LIN evaluated on ISIC 2017 validation set. For comparison convenience, the frameworks only take a single scale of lesion images, i.e., ~300 × 300, as input. As shown in Table 3, due to the overfitting problem, the JA of FCRN-88 trained with DR + DM is the lowest, i.e., 0.607. The proposed LIN achieves the best performance, i.e., 0.710.

##### Experiments on the Multi-Scale Input Images

Taking computation efficiency into account, the original skin lesion images were cropped and resized to 320 × 320 for network training. However, lesion images of larger scale (~500 × 500) provide a clearer view of the lesion area, e.g., the texture, for feature extraction. To demonstrate the importance of processing skin lesion images at multiple scales, a set of experiments were conducted. Three scales of testing images were selected, i.e., ~300 × 300, ~500 × 500 and ~700 × 700, for comparison. The comparison results are presented in Table 4.

For single scale, an input image of ~300 achieves the best performance on the ISIC validation set, i.e., a JA of 0.710. Degradation of segmentation performance is observed when only using the larger-scale images, i.e., degradations of 0.012 and 0.048 for ~500 and ~700, respectively. However, the larger-scale input images can assist LIN to perform more accurate segmentation. The LIN using all of three scales achieves the best JA, i.e., 0.753, which is 0.002 higher than the second-rank, i.e., LIN using ~300 and ~500. In consideration of computational efficiency, the LIN using ~300 and ~500 is preferable for experiments and applications.

#### 3.3.2. The Performance on Lesion Classification

##### Performance of LICU

Each pixel in the lesion images has different importance for the final classification result. Although the FCRN-88 can simultaneously perform segmentation and classification tasks, it assigns equal importance for all pixels. Lesion Index Calculation Unit (LICU) measures the pixel importance by distance map, and accordingly refines the possibility maps from FCRN-88s. Experiments were conducted on the ISIC 2017 validation set to assess the performance of LICU. Table 5 lists the results. Compared to the plain LIN, i.e., 0.891, the LICU component produces an improvement of 0.021 for LIN, i.e., 0.912.

### 3.4. Lesion Feature Network (LFN)

#### 3.4.1. Analysis of Network Architecture

To analyze the influence caused by layer width, we transform the original LFN to two variations for comparison, i.e., Narrow LFN and Wide LFN, the detailed information for which is listed in Table 6.

The performances of three LFNs were evaluated on ISIC 2017 validation set in Table 7. By comparing the AUC of LFN and Narrow LFN, we notice that the narrow layer decreases the capacity of feature representation of framework. The AUC of Narrow LFN is 0.822, which is 0.026 lower than that of LFN, i.e., 0.848. In another aspect, too wide a layer leads to the overfitting problem, which also decreases the performance of LFN. The AUC of wide LFN (0.803) is 0.045 lower than that of the original LFN. Hence, the proposed LFN better balances the relationship between feature representation capacity of framework and the network overfitting problem.

#### 3.4.2. Performance of Weighted Softmax Loss (WSL)

Although a data augmentation approach was used to balance the sample volumes of different categories, the generated training set is still imbalanced. Weighted softmax loss (WSL) is another important tool for alleviating the influence caused by an imbalanced training set during network training. As shown in Table 7, without using WSL, the AUC of LFN sharply decreases to 0.778, which demonstrates the importance of weighted softmax loss.

#### 3.4.3. Usage of Batch Normalization (BN)

Batch normalization (BN) [39] components can reduce internal covariate shift and accelerate the training process, and has been widely adopted in many deep learning frameworks, e.g., ResNet [33] and Inception [40]. In the proposed LFN, BN is adopted between convolutional layer and rectified linear units layer. The result presented in Table 7 indicates that an improvement of 0.006 is generated by a BN component for AUC.

## 4. Comparison with Benchmarks

To further evaluate the performance of proposed LIN and LFN, we compared them with several existing deep learning frameworks on ISIC 2017 validation set.

### 4.1. Lesion Segmentation

For lesion segmentation, the fully convolutional network (FCN) proposed by Long et al. [41], the U-net [42], the fully-convolutional Inception (II-FCN) [43] and the encoder-decoder network using RNN layer (Auto-ED) [44] are included for comparison. The results are listed in Table 8.

Although our LIN is the deepest network among the listed algorithms, the balanced data augmentation strategy and dual-network structure alleviate the overfitting problem. Table 8 shows that the proposed LIN achieved the best JA (0.753), AC (0.950) and DC (0.839) among the presented benchmark algorithms. The Auto-ED ranks second, i.e., 0.738, 0.936 and 0.824 were achieved for JA, AC and DC, respectively. The U-net and II-FCN produced the best SE (0.853) and SP (0.984), respectively.

### 4.2. Dermoscopic Feature Extraction

For the task of dermoscopic feature extraction, as little work has addressed it, only the framework proposed by Kawahara [45] was included for comparison. The results are shown in Table 9.

The framework proposed by Kawahara converted the problem of dermoscopic feature extraction to a semantic segmentation task, which was supervised by a revised F1 score. As the F1 score takes the overlapping area between prediction and ground truth as the main criterion for network training, Kawahara’s framework yields better performance on predicting the topological structure of dermoscopic features. As a consequence, it achieves higher AUC (0.893), AC (0.985) and SP (0.987) than that of the proposed LFN. Different from Kawahara’s framework, the proposed LFN is a patch-based classification network. It yields decent results on edge detection of dermoscopic features, which results in a higher average precision (AP) and sensitivity (SE), i.e., 0.422 and 0.693, than that of the framework ranking in second place.

### 4.3. Lesion Classification

Table 10 lists the lesion classification results of different frameworks, which includes AlexNet [46], VGG-16 [47], ResNet-50/101 [33] and Inception-v3 [40]. The proposed LIN achieved the best AUC (0.912), AC (0.857) and AP (0.729) among the presented benchmark algorithms, which are 0.02, 0.01 and 0.017 higher than the second ranks, respectively. The ResNet-50 and ResNet-101 produce excellent performances for SE (0.845) and SP (0.986), respectively. As the Inception-v3 is an extremely deep network, it easily encounters the overfitting problem and achieves relatively low AUC (0.800) and AP (0.564) among the benchmarking algorithms.

## 5. Conclusions

In this paper, we proposed two deep learning frameworks, i.e., the Lesion Indexing Network (LIN) and the Lesion Feature Network (LFN), to address three primary challenges of skin lesion image processing, i.e., lesion segmentation, dermoscopic feature extraction and lesion classification.

The Lesion Indexing Network was proposed to simultaneously address lesion segmentation and classification. Two very deep fully convolutional residual networks, i.e., FCRN-88, trained with different training sets, are adopted to produce the segmentation result and coarse classification result. A lesion indexing calculation unit (LICU) is proposed to measure the importance of a pixel for the decision of lesion classification. The coarse classification result is refined according to the distance map generated by LICU.

The Lesion Feature Network was proposed to address the task of dermoscopic feature extraction, and is a CNN-based framework trained by the patches extracted from the dermoscopic images. To the best of our knowledge, we are not aware of any previous work available for this task. Hence, this work may become a benchmark for subsequent related research.

Our deep learning frameworks have been evaluated on the ISIC 2017 dataset. The JA and AUC of LIN for lesion segmentation and classification are 0.753 and 0.912, which outperforms the existing deep learning frameworks. The proposed LFN achieves the best average precision and sensitivity, i.e., 0.422 and 0.693, for dermoscopic feature extraction, which demonstrates its excellent capacity for addressing the challenge.

## Figures and Tables

**Table 1:** Detailed information of data augmentation (task 1 & 3).

	Melanoma (18°)	Seborrheic Keratosis (18°)	Nevus (45°)
Original	374	254	1372
DR	7480	5080	10,976

**Table 2:** Detailed information of data augmentation (task 2).

	Original	Random Sample + Rotation
Background (B)	>90,000	87,089
Pigment Network (PN)	>80,000	77,325
Negative Network (NN)	~3000	12,908
Milia-like Cysts (MC)	~5000	18,424
Streaks (S)	~2000	8324

**Table 3:** JA of frameworks on ISIC 2017 validation set.

Model	JA
FCRN-88 (DR)	0.697
FCRN-88 (DR + DM)	0.607
LIN (ours)	0.710

**Table 4:** JA of frameworks with different scales of inputs.

Model	*JA*
LIN (~300)	0.710
LIN (~500)	0.698
LIN (~700)	0.662
LIN (~300 + ~500)	0.751
LIN (~300 + ~500 + ~700)	0.753

**Table 5:** AUC of frameworks with/without LICU.

Model	AUC
LIN without LICU	0.891
LIN with LICU	0.912

**Table 6:** Detailed information of different LFNs

	LFN	Narrow LFN	Wide LFN
Stage 1	16, (3,3)	16, (3,3)	32, (3,3)
	16, (1,1)	16, (1,1)	32, (1,1)
	16, (3,3)	16, (3,3)	32, (3,3)
Stage 2	32, (3,3)	16, (3,3)	64, (3,3)
	32, (1,1)	16, (1,1)	64, (1,1)
	32, (3,3)	16, (3,3)	64, (3,3)
Stage 3	64, (3,3)	16, (3,3)	64, (3,3)
	64, (1,1)	16, (1,1)	64, (1,1)
	64, (3,3)	16, (3,3)	64, (3,3)
Stage 4	128, (3,3)	32, (3,3)	128, (3,3)
	128, (1,1)	32, (1,1)	128, (1,1)
	128, (3,3)	32, (3,3)	128, (3,3)

**Table 7:** AUC of LFNs on the validation set.

Model	AUC
Narrow LFN	0.822
Wide LFN	0.803
LFN	0.848
LFN (without WSL)	0.778
LFN (without BN)	0.842

**Table 8:** Lesion segmentation performances of different frameworks.

Method	JA	AC	DC	SE	SP
FCN-8s [41]	0.696	0.933	0.783	0.806	0.954
U-net [42]	0.651	0.920	0.768	0.853	0.957
II-FCN [43]	0.699	0.929	0.794	0.841	0.984
Auto-ED [44]	0.738	0.936	0.824	0.836	0.966
LIN (ours)	0.753	0.950	0.839	0.855	0.974

**Table 9:** Dermoscopic feature extraction performances of different frameworks.

Method	AUC	AC	AP	SE	SP
J. Kawahara [45]	0.893	0.985	0.185	0.534	0.987
LFN (ours)	0.848	0.902	0.422	0.693	0.902

**Table 10:** Lesion classification performances of different frameworks.

Method	AUC	AC	AP	SE	SP
AlexNet [46]	0.859	0.823	0.651	0.343	0.969
VGG-16 [47]	0.892	0.847	0.709	0.586	0.919
ResNet-50 [33]	0.873	0.723	0.690	0.845	0.694
ResNet-101 [33]	0.869	0.840	0.712	0.336	0.986
Inception-v3 [40]	0.800	0.800	0.564	0.355	0.933
LIN(Ours)	0.912	0.857	0.729	0.490	0.961
